# Treatment of Chronic Atrial Fibrillation During Surgery for Rheumatic Mitral Valve Disease

**DOI:** 10.5935/1678-9741.20160070

**Published:** 2016

**Authors:** Flavio Donizete Gonçalves, Valdir Gonçalves Leite, Vanusa Gonçalves Leite, Marcelo Alves Maia, Otoni Moreira Gomes, Melchior Luiz Lima, Evandro César Vidal Osterne, Elias Kallás

**Affiliations:** 1 Hospital Aroldo Tourinho, Montes Claros, MG, Brazil.; 2 Fundação Cardiovascular São Francisco de Assis - ServCor- Belo Horizonte, MG, Brazil.

**Keywords:** Rheumatic Heart Disease, Heart Valves, Mitral Valve, Atrial Fibrillation

## Abstract

**Introduction:**

The result of surgical ablation of atrial fibrillation remains controversial, although prospective and randomized studies have shown significant differences in the return to sinus rhythm in patients treated with ablation *versus* control group. Surgery of the Labyrinth, proposed by Cox and colleagues, is complex and increases the morbidity rate. Therefore, studies are needed to confirm the impact on clinical outcomes and quality of life of these patients.

**Objective:**

To analyze the results obtained in the treatment of atrial fibrillation by surgical approach, by Gomes procedure, for mitral valve surgery in patients with rheumatic heart disease associated with chronic atrial fibrillation.

**Methods:**

We studied 20 patients with mitral valve dysfunction of rheumatic etiology, evolving with chronic atrial fibrillation, submitted to surgical treatment of valvular dysfunction and atrial fibrillation by Gomes procedure.

**Results:**

The mean duration of infusion ranged from 65.8±11.22 and aortic clamping of 40.8±7.87 minutes. Of 20 patients operated, 19 (95%) patients were discharged with normal atrial heart rhythm. One (5%) patient required permanent endocardial pacing. In the postoperative follow-up of six months, 18 (90%) patients continued with regular atrial rhythm, one (5%) patient returned to atrial fibrillation and one (5%) patient continued to require endocardial pacemaker to maintain regular rhythm.

**Conclusion:**

Gomes procedure associated with surgical correction of mitral dysfunction simplified the surgical ablation of atrial fibrillation in patients with rheumatic mitral valve disease and persistent atrial fibrillation. The results showed that it is a safe and effective procedure.

**Table:** 

Abbreviations, acronyms & symbols	
AFFIRM	=Atrial Fibrillation Follow-up Investigation of Rhythm Management
CPB	=Cardiopulmonary bypass
ICU	=Intensive care unit
INR	=International normalized ratio

## INTRODUCTION

Due to the lack of knowledge of electrophysiological mechanisms of atrial fibrillation, for a long time, reduction of ventricular rate was the only existing treatment for the reduction of symptoms. Pharmacological treatment of this disease relies on the use of antiarrhythmic drugs. In patients with risk factors for thromboembolic events, anticoagulants are associated. However, in medium and long-term, drug therapy is unable to prevent the recurrence of atrial fibrillation outbreaks in 50% of patients, which leads to significant loss of life quality^[1]^.

Several studies attested such claims, particularly the multicenter study "Atrial Fibrillation Follow-up Investigation of Rhythm Management" (AFFIRM)^[2]^, which reported that the antiarrhythmic drug therapy for maintenance of sinus rhythm was not beneficial when compared to the ventricular rate control associated with anticoagulation in relation to mortality and ischemic stroke.

Pharmacological clinical treatments of atrial fibrillation, in addition to not correcting it and being costly, has significant morbidity and mortality, justifying the urgent need for invasive alternative treatment, whether surgical or percutaneous^[3]^.

The surgical alternative to atrial fibrillation treatment became a reality from studies of Haissaguerre et al.^[4]^, which highlighted the key role of the pulmonary veins in the pathophysiology of atrial fibrillation episodes.

In Brazil, rheumatic heart disease still has a high prevalence. Its structural sequelae represent one of the main causes of heart valve surgery. In this context, atrial fibrillation of rheumatic nature, obviously, remains a major medical and surgical problem^[5]^.

In patients with mitral valve disease and atrial fibrillation, surgical correction of valvular dysfunction does not result generally in solution for arrhythmia, because recurrence rates are high, reaching up to 80% in six months^[6]^.

Coumel et al.^[7]^, performed the first surgery for the treatment of ectopic foci of arrhythmia located in the left atrial, leading to the subsequent development of left atrial isolation techniques in the treatment of atrial fibrillation.

The evolution of atrial fibrillation surgery included the development of less invasive surgical techniques, by replacing the section lines and atrial sutures by the use of energy sources in the atrial myocardium. The goal was to create transmural lesions to block the macro reentry circuits. The main energy sources currently employed are cryothermia, radiofrequency, microwave, ultrasound and laser beams^[5]^.

The results of surgical ablation of atrial fibrillation remains controversial, although prospective and randomized studies have shown significant differences in the return to sinus rhythm in patients treated with ablation *versus* control group. However, further studies are needed to confirm the impact on clinical outcomes and quality of life of these patients^[8]^.

The innovative highlight was the publication of Gomes & Gomes^[9]^, who described pioneering surgical procedure of simultaneous correction of mitral valve disease associated with atrial fibrillation.

This study aims to present the results of Gomes procedure in randomly consecutive patients with rheumatic mitral valve disease and chronic atrial fibrillation.

## METHODS

From June 2006 to March 2013, 20 patients, 15 (75%) female gender and 5 (25%) male, with ages between 20-65 years (mean 44.25±13.9) carriers of rheumatic mitral valve disease and chronic atrial fibrillation (lasting at least 1 year) were operated at the Cardiovascular Surgery Service of the Hospital Aroldo Tourinho, Montes Claros - Minas Gerais, Brazil (Table 1). The study was approved by the Research Ethics Committee of the Hospital Aroldo Tourinho.

**Table 1:** Demographic data of patients.

Patient	*Age (years)	Gender
1	57	M
2	45	F
3	53	F
4	65	M
5	24	F
6	42	M
7	38	M
8	51	F
9	27	F
10	31	F
11	59	F
12	52	F
13	20	F
14	50	F
15	37	F
16	31	F
17	33	F
18	65	F
19	44	F
20	61	M

* Mean 44.25±13.78 F=female; M=male

The symptoms related to mitral valve disease were the reason to the surgical indication in all patients. None of the patients was operated on an emergency basis. All patients underwent preoperative echocardiography to study heart valves and analysis of intra cavity thrombi in addition to evaluate parameters such as left atrium diameter, left ventricular ejection fraction, valve area and right chambers pressure.

Cardiac catheterizations were performed in patients aged greater than or equal to 40 years, not revealing obstructions in the coronary arteries in any of them.

Inclusion criteria were patients with rheumatic mitral valve disease and chronic atrial fibrillation.

The exclusion criterion was the need for other associated surgical procedures.

The following data from records collected on the day of surgery were considered to study: cardiopulmonary bypass time, aortic clamping time, sinus rhythm correction and postoperative bleeding.

The cardiac rhythm was evaluated in four different times: after release of the aortic clamp, in the intensive care unit (ICU), at hospital discharge and at the end of the postoperative follow-up.

All patients received amiodarone 200 mg/day and sodium warfarin 5 mg/day adjusted as international normalized ratio (INR) of prothrombin activity for 3 months.

The surgical technique included the use of normothermic cardiopulmonary bypass (36-37.5ºC) with total venous drainage through cannulas inserted in the upper and lower vena cava, through areas bounded by sutures in bags in the right atrium. The suture of the tube from the superior vena cava was placed 5 mm from the base of the right atrium appendage.

The cannula for systemic arterial perfusion was inserted into the distal portion of the ascending aorta. The cannula for coronary arterial cardioplegic perfusion was inserted into the proximal portion of the aorta. Myocardial protection was achieved with blood coronary perfusion anterograde hyperkalemic (25 mEq/l), hypothermic (4°C), with perfusion pressure of 60-90 mmHg and intermittent. It was repeated every 20 to 25 minutes.

**Fig. 1:**
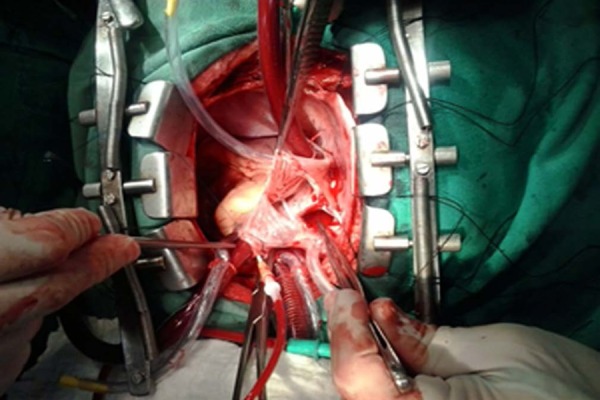
Purse string suture at the base of the right atrium appendage and biatrial incision to section the atrial septum.

To access the mitral valve, atrial ostia of the pulmonary veins and the left atrial appendage, as well to section the atrial septal and right atrial conduction areas, a single oblique incision was used. This incision was started 5 mm above the right atrioventricular groove and extended up to 15 mm in the anterior contour of the right superior pulmonary vein with section of the atrial septum up to 10 mm above the annulus of the tricuspid valve (Figure 1).

Thrombi in the left atrium, present in 20% of cases, were removed.

**Fig 2:**
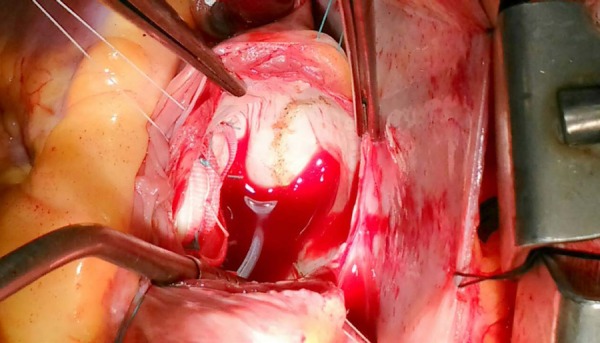
Left atrium open revealing line of cauterization of the pulmonary vein ostia.

Compartmentalization of the left atrium was performed with the use of electrocautery, isolating the pulmonary vein ostia (Figure 2).

The left atrial appendage was excluded by continuous suture from its base in the communication ostium with the left atrium.

**Fig.3:**
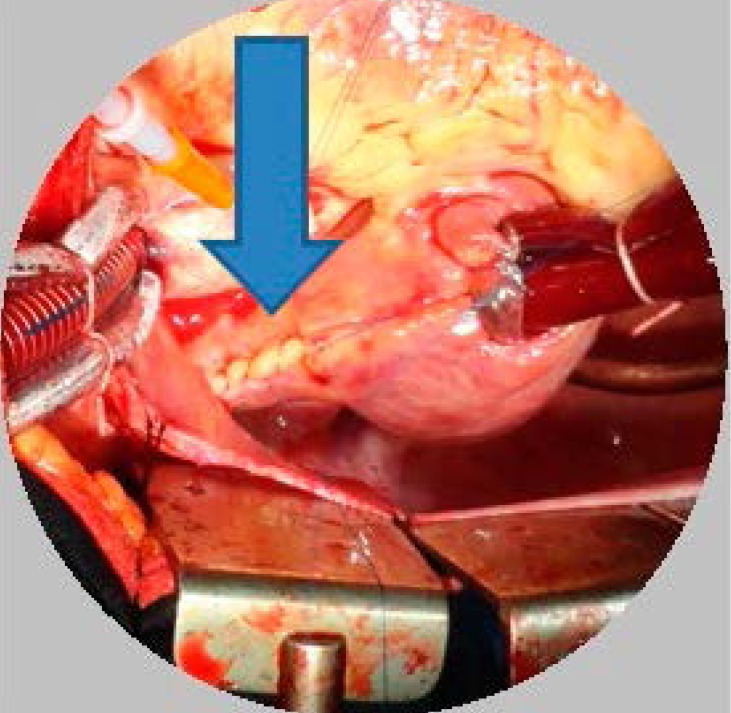
Longitudinal suture in the anterior contours of the superior vena cava (arrow).

The circuits of anomalous focus of cardiac stimulation, in the superior and inferior vena cava were interrupted by longitudinal sutures, each one measuring 1 cm in length (Figure 3).

The reconstruction of the atrial septum, the right superior pulmonary vein and the right atrial wall, continuous sutures were used in single plane with 3-0 polypropylene thread. The right atrium auricle was excluded by simply closure of purse string suture after removal of the superior vena cava cannula.

## RESULTS

In 18 patients, mitral valve replacement was performed by pericardial bioprosthesis, 10 units from the Labcor^®^, 6 units from Braile^®^ and 2 units from Saint Jude^®^ In 2 patients, the mitral valve was preserved, commissurotomy being held in one patient and valvuloplasty with mitral repair ring Carpentier type (Labcor^®^) in another patient.

The left atrial size ranged from 4.5 to 6.0 cm and 5 patients had thrombi in the atrium and left atrial appendage. The extracorporeal perfusion time ranged 43-88 minutes averaging 65.8 minutes, and the aortic clamping time ranged 22-50 minutes with an average of 40.8 minutes.

There was no mortality in patients studied in the six months follow-up. In one (5%) patient was necessary dual-chamber pacemaker implantation because of symptomatic sinus bradycardia, and this patient already had atrial fibrillation with pauses longer than 2 seconds before the operation.

No patient had hemorrhagic or thromboembolic complication during the study follow-up period (Table 2).

**Table 2:** Postoperative bleeding and blood transfusion.

Patient	Blood transfusion	Bleeding (ml)	
		1^st^hour	24^rd^hour
1	Yes	280	550
2	No	100	400
3	No	125	300
4	Yes	15	150
5	Yes	60	1000
6	No	15	150
7	No	100	300
8	No	100	200
9	No	20	70
10	Yes	200	600
11	No	50	125
12	No	75	150
13	No	50	140
14	No	40	300
15	Yes	180	400
16	No	20	150
17	No	50	300
18	No	100	450
19	Yes	125	550
20	No	75	350

In all patients, we obtained regular atrial cardiac rhythm, with hemodynamic stabilization at the end of cardiopulmonary bypass. Thus, 19 patients were released from ICU and later discharged with sustained regular atrial heart rhythm.

One (5%) patient required endocardial dual-chamber pacemaker implantation because of symptomatic sinus bradycardia (Table 3).

**Table 3:** Perioperative results.

Patient	Cardiopulmonary bypass time (min)	Aortic clamping time (min)	Procedure	Diameter (mm)
1	69	45	Valve replacement	29
2	61	43	Valve replacement	31
3	50	37	Valve replacement	29
4	70	32	Valve replacement	33
5	66	50	Valve replacement	31
6	57	38	Valve replacement	31
7	75	41	Valve replacement	29
8	66	55	Valve replacement	29
9	77	50	Valve replacement	29
10	88	44	Valve replacement	27
11	79	51	Valve replacement	29
12	69	40	Valve replacement	29
13*	70	46	Valve replacement	27
14	57	34	Valve replacement	29
15	47	22	Commissurotomy	-
16	43	30	Valve replacement	27
17	59	42	Valve replacement	31
18	75	40	Valve replacement	29
19	70	40	Valve replacement	29
20	69	36	Annuloplasty	30

* Pacemaker

All patients were discharged in stable clinical conditions, with 19 patients in regular rhythm with presence of the P wave and one patient in dual chamber pacemaker rhythm.

In the postoperative six months follow-up, 18 (90%) patients continued with regular atrial rhythm, one (5%) patient returned to atrial fibrillation and one patient presented with sinus bradycardia, requiring endocardial pacemaker implantation (5%).

During hospitalization and 12 weeks after surgery, 19 patients received amiodarone 200 mg daily and Warfarin sodium as INR.

## DISCUSSION

Atrial fibrillation is a supraventricular arrhythmia of higher clinical interest, not only for its high incidence, but also by the possibility of developing severe cardiovascular disorders, such as stroke or heart failure^[10]^.

Its prevalence may vary from 0.15% to 1%, increasing gradually with age. In individuals older than 62 the prevalence can reach 5% to 9%^[11]^. With the growing interest in understanding the pathophysiology responsible for the occurrence of this arrhythmia, operative techniques have been developed in order to increase the effectiveness of this operation and reduce the possibility of treatment failure, which can reach 20% of patients undergoing operative procedures for treatment of arrhythmia in association with mitral valve management^[12]^.

Developed over 10 years, the Maze operation is still considered the reference method for the surgical treatment of atrial fibrillation. However, the routine use of this surgery is limited to a few centers, given its complexity.

Several studies have shown that in controlling atrial fibrillation, the mere creation of lines by cutting and suturing or a radiofrequency ablation, contouring or uniting the ostia of the pulmonary veins determines clinical results similar to those obtained by the Maze procedure^[6]^.

Different surgical techniques have been proposed with the common point of targeting interventions to the posterior wall of the left atrium, more specifically for the region of the pulmonary veins. Jatene et al.^[13]^ performed the Cox surgery for treatment of atrial fibrillation in 45 patients with rheumatic mitral valve disease due to its usual occurrence in our country, but in this study instead of Cox surgery, we used the technique reported by Gomes & Gomes^[9]^ also to treat rheumatic mitral valve disease associated with atrial fibrillation.

In Brazil, Jazbik et al.^[14]^, have proposed different surgical techniques for the management of atrial fibrillation, and its common point would be the left atrium reduction by excision of atrial tissue bands. Vasconcelos et al.^[15]^ performed a study to evaluate the effectiveness of surgical isolation of the left atrial posterior wall involving the ostia of the pulmonary veins for the treatment of atrial fibrillation in patients with rheumatic mitral valve disease.

Gomes & Gomes^[9]^ technique replaces the resection of the right and left atrial appendages. It uses a purse string suture, in the introduction of the cannula in the superior vena cava in the right atrium and by the closing of the ostium of the left atrial appendage by intra-atrial suture respectively; and facilitates to a broad internal exposure of the left atrium by the single atrial incision.

From the electrophysiological point of view, a single incision in the right atrium excludes one of the stimuli of reentry circuits in the atrial wall and also the septal channels. The circuits near the ostia of the superior and inferior vena cava veins were considered by Frame et al.^[16]^ as capable of generating tachyarrhythmias. Interruption can be performed easily by applying a simple suture of 1 cm in length in the anterior border of the vena cava.

Brick et al.^[5]^ report's approach carried out along the lines described by Haissaguerre et al.^[4]^, done with the application of radiofrequency so it decreased the duration of CPB, together with all its benefits.

Cox operation, carried out in Brazil by Jatene et al.^[13]^, showed excellent results in the reversal of atrial fibrillation, particularly in heart valve disease to sinus rhythm. However, as described by Cox et al.^[17]^, which highlighted the prolonged times of CPB, these authors cite as limiting the long time of operation when performed by less experienced professionals is perhaps a limiting factor for the spread of the technique.

The CPB time and smaller aortic clamping are very important, because both are associated with the risk of complications in surgery. In the present study the CPB time ranged from 43-88 minutes (65.8±11.2) and the aortic clamping time 22-50 minutes (40.8±7.9); shorter than that obtained in the Cox surgery even in referral centers.

Cox et al.^[17]^ compartmentalized the right and left atria through the section of the walls and suturing in lines for the purpose of disrupting micro entries; while Gomes Júnior et al.^[18]^, used the left atrial longitudinal incision. In this study, we used the single oblique atrial incision in the right atrium and the atrial septum extending to the right superior pulmonary vein, causing section of interatrial and septal pathways.

In the Vasconcelos et al.^[15]^ study, the inclusion of a control group permitted the establishing of the isolation of the posterior wall of the left atrium, encompassing the ostia of the pulmonary veins, used concomitantly with the valvular surgical treatment in patients with chronic rheumatic heart disease. It is a safe and effective procedure in the treatment of atrial fibrillation, promoting a reduction in the incidence of recurrence of arrhythmia, both in the perioperative phase in late stage.

Saad & Camanho^[19]^ report that the selection of patients for radiofrequency ablation procedure with persistent or permanent forms of atrial fibrillation follows the same reasoning, but the decision should be individualized according to the duration of atrial fibrillation and the size of theleft atrium, an important predictor of recurrence. Even with extensive applications of radiofrequency, the rate of recurrence and the need for new procedures are higher in this group, reaching 40% of cases.

Kosakai et al.^[20]^, in a series of 62 mitral valve disease patients, succeeded in controlling atrial fibrillation in 84% of them, expanding more recently the series and keeping the same results. These authors attribute the increased size of the left atrium the failure registered in about 10% of the patients, who did not obtain control of atrial fibrillation. In Jatene et al.^[13]^, experience where the average size of the left atrium was 5.5 cm, it was possible to control atrial fibrillation in 90% of cases, including people with different valve disease, including the valve reoperation. In 2 patients who remained in a long-term atrial fibrillation, the left atrium measured about 6.0 cm, which was perhaps one of the main factors for the failure of the operation. The left atrial size ranged from 4.5 to 6.0 cm and did not interfere in the atrial fibrillation correction results in patients in this study.

Gomes & Gomes^[9]^ obtained regular atrial rhythm with hemodynamic stabilization at the end of CPB in all patients.

The results observed by Jatene et al.^[13]^, using the Cox technique showed cardioversion of atrial fibrillation to regular rhythm in all cases after CPB, with maintenance of the results in a short and long term follow-up by 95%.

Lins et al.^[21]^ report that the cardioversion of atrial fibrillation to sinus rhythm in the immediate postoperative period was 20 (90.9%) from 22 patients in the group receiving ablation *versus* 3 (13.6%) of the 22 patients group which were not submitted to ablation.

In the Brick et al.^[5]^ series of cases study was observed reversal from atrial fibrillation rhythm to sinus rhythm in 24 (88.8%) of the 27 patients in the immediate postoperative period and was 22 (81.4%) at hospital discharge.

In this study, there was a reversal to sinus rhythm in all patients at the end of CPB. However, one patient with symptomatic sinus bradycardia required a definitive endocardial pacemaker implantation. In cases of mitral valve disease associated with atrial fibrillation, the use of Gomes & Gomes^[9]^ technique in association with treatment of valve disease, there can be observed significant clinical improvement of patients in the late postoperative period. This situation was also observed in reports of Kosakai et al.^[20]^.

Calkins et al.^[22]^, observed reversion to sinus rhythm in 28% of left atrium after intervention on mitral valve showing correlation of the diameter of the left atrium (left atrium > 52 mm) with the maintenance of atrium fibrillation despite the effective treatment of valve disease mitral.

In this study, 19 patients were discharged from hospital in regular sinus rhythm and 1 patient required endocardial pacing due to sinus bradycardia.

Jatene et al.^[13]^, found that low cardiac output was a complication observed in 5 patients. Kosakai et al.^[20]^ used the intra-aortic balloon pump in 4 patients with low cardiac output. Sandoval et al.^[23]^, observed one case of a low cardiac output followed by death.

Kosakai et al.^[20]^ found an incidence of reoperation around 8% in the group of patients who underwent surgical treatment of atrial fibrillation, all with mitral valve disease.

Despite being a surgical procedure that requires careful surgical technique with bleeding potential this was not a complication observed in this study.

The embolic phenomena in the preoperative period of patients with mitral valve disease although more often associated with atrial fibrillation, also present a significant incidence in patients with sinus rhythm.

Boersma et al.^[24]^ reported a lower incidence of stroke and thromboembolism in surgical ablation when compared with catheter ablation in follow-up of 12 months. These findings are due to the fact that in the surgical procedure the thrombus in the left atrium can be easily removed during surgery.

In this study, although the occurrence of thrombi in the left atrial appendage has happened in 20% of patients, there were no cases of stroke or embolism during the follow-up period, probably due to direct removal of thrombus in the perioperative term.

Canale et al.^[25]^ report that surgical mortality of 13% reflects the gravity and the late stage of the disease in these patients which are referred for surgery and considering the presence of atrial fibrillation demonstrates an advanced mitral disease.

Gomes & Gomes^[9]^, as well as Stulak et al.^[10]^, report that there was no mortality in the patients undergoing surgery.

Stulak et al.^[10]^, in 2006, reported that three patients in the studied group (37 patients) required a permanent pacemaker due to sinus node disease.

In this study, along the 6 months follow-up there was no mortality. One (5%) patient required permanent pacemaker implantation, and this patient already had pauses preoperatively with pacemaker indication prior to correction of the atrial fibrillation.

## CONCLUSION

The consistent results of this study demonstrate that Gomes technique is a safe and effective therapeutic procedure for the treatment of chronic atrial fibrillation in surgery of patients with rheumatic mitral valve disease.

**Table:** 

Authors’ roles & responsibilities	
FDG	Conception and design study; realization of operations and/or trials; manuscript writing or critical review of its content; final manuscript approval
VGLJ	Conception and design study; realization of operations and/or trials; manuscript writing or critical review of its content; final manuscript approval
VGL	Conception and design study; realization of operations and/ or trials; analysis and/or data interpretation; final manuscript approval
MAM	Conception and design study; manuscript writing or critical review of its content; final manuscript approval
OMG	Conception and design study; manuscript writing or critical review of its content; final manuscript approval
MLL	Analysis and/or data interpretation; statistical analysis; final manuscript approval
ECVO	Analysis and/or data interpretation; statistical analysis; final manuscript approval
EK	Analysis and/or data interpretation; manuscript writing or critical review of its content; final manuscript approval
